# Exploring nurses’ perception about the care needs of patients with COVID-19: a qualitative study

**DOI:** 10.1186/s12912-020-00516-9

**Published:** 2020-12-11

**Authors:** Nasrin Galehdar, Tahereh Toulabi, Aziz Kamran, Heshmatolah Heydari

**Affiliations:** 1 Social Determinates of Health Center, Lorestan University of Medical Sciences, Khorramabad, Iran; 2 School of Nursing and Midwifery, Lorestan University of Medical Sciences, Khorramabad, Iran; 3 School of Medicine and Allied Medical Sciences, Ardabil University of Medical Sciences, Ardabil, Iran; 4 Social Determinates of Health Center, Lorestan University of Medical Sciences, Khorramabad, Iran; 5 French Institute of Research and High Education (IFRES-INT), Paris, France

**Keywords:** COVID-19, Nurses, Patients, Caring needs, Qualitative research

## Abstract

**Background:**

COVID-19 is a new disease affecting and killing a large number of people across the world every day. One way to improve health care for these patients is to recognize their needs. Nurses, as a large population of health care staff, can be rich sources of information and experience on patients’ care needs. Therefore, the aim of this study was to explore nurses’ perception about the care needs of patients with COVID-19.

**Methods:**

The present qualitative research was performed using the conventional content analysis approach in Iran from March to May 2020. The participants of this study included the nurses caring for patients with COVID-19, recruited by the purpose sampling method. The data was collected through 20 telephone interviews and analyzed based on the method proposed by Lundman and Graneheim.

**Results:**

Qualitative data analysis revealed six main categories including need for psychological consulting, need for quality improvement of services, need for upgrading of information, need for improving of social support, need for spiritual care and need for social welfare.

**Conclusion:**

The data showed that patients with COVID-19 were psychologically, physically, socially, economically, and spiritually affected by the disease. Therefore, they should be comprehensively supported by health care staff and other supportive systems.

## Background

COVID-19 is a newly emerged infectious disease which was first reported in Wuhan, China on December 31, 2019 [1]. After a rapid spread which inflicted many countries across the world, the disease was declared as a pandemic by the World Health Organization (WHO) on March 11, 2020 [2]. Up until May 25, 2020, the global number of people contracting the COVID-19 and the death toll had reached 5,131,810 and 331,108, and in Iran, these numbers were 129,341 and 7249, respectively [3]. Around 20% of patients with the infection may experience severe symptoms requiring oxygen therapy or other inpatients interventions, and only 5% of these will require hospitalization in the intensive care unit (ICU) [4].

Studies on patients with the COVID-19 indicate that they may experience various symptoms such as fever, dyspnea, muscle ache, headache, fear, diarrhea, nausea, vomiting, increased systolic blood pressure, and hemoptysis, that require invasive and non-invasive therapeutic supports during the acute course of the disease [5, 6]. The mortality rate of COVID-19 has been estimated as 1 to 5%, but this varies based on patients’ age groups and the presence or absence of underlying diseases [7]. Previous experiences of the SARS crisis indicated that patients may face many problems such as fear, loneliness, boredom, anger, anxiety, insomnia, and feeling of a taboo. Also, patients may have concerns regarding the effects of quarantine on their psychological well-being and the risk of infecting family members and friends [8]. The COVID-19, as a SARS-related emerging disease [9], has many unknown dimensions in various clinical care areas.

For a comprehensive patient care, their needs should be identified. Among health care providers, nurses are at the forefront of fighting against the COVID-19. They are in close and constant contact with patients from admission to discharge. Also, nurses are valuable resources to recognize the patients’ needs, clinical manifestations of the disease, evidence-based care practices, nursing management problems, and prognostic factors during the COVID-19 crisis [10]. Explaining nurses’ perception of COVID-19 patients’ needs can be helpful to improve the quality of patient care. A few studies have been carried out on nurses’ experiences about the caring needs of patients with COVID-19. Because of uncertainties about the diverse aspects of the disease and caring needs of patients, and the fact that the authors are proficient in qualitative research methodology, and because they are closely engaged with caring of patients with COVID-19, the aim of this study was to use a qualitative research approach to explore nurses’ perception about the care needs of COVID-19 patients. The results of this study can be helpful in improving the quality of care for patients with COVID-19.

## Methods

This qualitative study was performed with a conventional content analysis approach.

### Participants

The study population included the nurses occupied in the inpatient wards of COVID-19 of general hospitals affiliated to Lorestan University of Medical Sciences. The participants were selected using the purposeful sampling method based on the length of work experience in COVID-19 wards, total years of work experience, the wards where the nurses were occupied before the COVID-19 crisis, and the participants’ age and marital status. The inclusion criteria were being engaged with caring for COVID-19 patients, willingness to participate in the study, and having at least 2 weeks of working experience in COVID-19 wards. The exclusion criterion was withdrawal from the study for any reason.

### Collecting data

Given the need for urgent data collection to improve the quality of patient care as well as the restrictions of face-to-face interview, the data was gathered through in-depth semi-structured telephone interviews from March to May, 2020. The characteristics of the nurses occupied in COVID-19 wards were initially obtained by referring to the nursing officials of the hospitals providing care for these patients. Then the participants were selected based on the snowballs theoretical approach. After explaining the objectives of the study and acquiring verbal consent for participation, an appropriate time was agreed for the interview. All the interviews were recorded by an electronic device. The main questions of the study were: “How would you describe a day of caring for hospitalized patients with COVID-19?”, and “What types of care do these patients need?”. Then the interview continued based on the participants’ answers with more detailed questions such as “Would you please explain more about this?”. Of course, the questions differ somewhat based on the position of the participant (i.e. head nurse, care provider, etc.) and the unit where the nurse was occupied (i.e. general or critical care unit). Using probing questions during the interview, the interviewer guided the process to achieve the study’s objectives.

### Data analysis

Data analysis was conducted simultaneously with the interviews based on the methods proposed by Lundman and Graneheim [11]. Detail of data analysis mentioned in the another article published of this study [12].

### Trustworthiness

For ensuring of the accuracy and reliability of the data, the criteria of credibility, dependability, transferability, and confirmability were used as proposed by Lincoln and Goba [13]. Detail of trustworthiness of data mentioned in the another article published of this study [12].

### Ethical considerations

Detail of ethical consideration mentioned in the another article published of this study [12].

## Results

In this study, a total of 20 nurses including 5 men and 15 women with an average age of 31.95 ± 6.64 years and a mean work experience of 7.25 ± 5.9 years were enrolled (Table 1). Data analysis in this study led to the emergence of six categories and 12 sub-categories (Table 2).

**Table 1:** Participants’ characteristics

Participant’s code	Sex	Level of education	Work experience (years)	Ward	Marital status	Position
1	Female	Post-graduate	4	Emergency	Single	Nurse
2	Female	Bachelor	22	CCU	Married	Head Nurse
3	Female	Post-graduate Student	10	CCU	Married	Nurse
4	Male	Post-graduate Student	7	Emergency	Married	Nurse
5	Female	Bachelor	20	ICU	Married	Nurse
6	Male	Post-graduate student	3	Emergency	single	Nurse
7	Female	Post-graduate student	4	Infectious	Single	Nurse
8	Female	Post-graduate	4	Emergency	Married	Nurse
9	Female	Bachelor	1	Emergency	Single	Nurse
10	Female	Bachelor	2	Emergency	Single	Nurse
11	Female	Bachelor	10	ICU	Married	Nurse
12	Female	Bachelor	8	ICU	Married	Nurse
13	Female	Bachelor	14	ICU	Married	Head Nurse
14	Female	Bachelor	4	General	Married	Nurse
15	Female	Post-graduate student	6	General	Married	Nurse
16	Male	Bachelor	4	ICU	Married	Nurse
17	Female	Bachelor	3	ICU	Single	Nurse
18	Female	Bachelor	12	ICU	Single	Nurse
19	Male	Bachelor	5	ICU	Single	Nurse
20	Male	Bachelor	2	ICU	Single	Nurse

**Table 2:** The categories and sub-categories extracted after data analysis

Categories	Sub-categories
Need for psychological consulting	Death anxiety
	Social stigma
	Hopelessness
	Separation anxiety
Need for quality improvement of services	Physical care
	Necessity for nutritional therapy
	Orientation
	Necessity for isolating of critically ill patients
Need for upgrading of information	Necessity for improving awareness and fighting superstition
	Institutionalizing a disease prevention culture
Need for improving of social support	Provision of familial communications
	Provision of personal accessories
Need for spiritual care	
Need for social welfare	

### Need for psychological consulting

Qualitative content analysis of the data showed that COVID-19 patients might suffer from many mental disorders and experience a lot of fear and panic during and after the disease. Therefore, they particularly need psychological consulting. Within this category, there were four sub-categories including death anxiety, social stigma, hopelessness, and separation anxiety.

#### Death anxiety

Data analysis showed that the patients equated being infected with COVID-19 to death and therefore were highly afraid of it. A participant noted that “... the atmosphere was like that the patients really had the perception that they would die of the disease, and there was no returning back ... “ *(p*_*17*_*).* The results also showed that the patients were horrified by the sudden death due to the failure of vital organs. Accordingly, one participant stated: “.... there might be nothing notable in the patient’s clinical status, but after a while, he/she would die after a reduction in O_2_ saturation ...” *(p*_*15*_*).* Based on the participants’ experiences, one of the reasons for the great fear of death was a restricted burial ceremony for the victims. In this regard, one of the participants said: “... well, I say that the condition is not good because they are not buried now, and they will not be buried on good terms ...” *(p*_*14*_*).*

#### Social stigma

According to the data analysis, one of the causes of patients’ anxiety was an impression to have a taboo disease. Data analysis showed that the patients with COVID-19 might perceive the disease as a social stigma and be ashamed of it. One participant described this as “... currently, all people have a bad view on COVID-19 patients....” *(p*_*14*_*).* Another participant mentions: “.... the stigma marked on the patients bothers them....” *(p*_*15*_*).*

#### Hopelessness

Data analysis showed a feeling of frustration in patients with the COVID-19 disease. One of their basic needs was to give them hope for life and the future. According to the participants, emotional support is one of the main patients’ primary needs. The participants also believed that patients with good mood would successfully recover from the acute phase of the disease. One of the participants, referring to the patients’ needs based on the Maslow pyramid, said: “.... I think the first need of ill patients is oxygen, ...... but those who have better condition need affection, or as Maslow said; the feeling of being belonged … “ *(p*_*14*_*).* Another participant shared the experience as: “ ... for example, we had a COVID-19 patient, a 23-year-old leukemic girl. We gave her hope as much as we could ... to the extent that she could really defeat the virus ... “ *(p*_*20*_*).*

#### Separation anxiety

Data analysis showed that COVID-19 patients would experience a difficult time during isolation due to physical problems, loneliness, being separated from the family, and the lack of a definitive treatment for the disease. The participants’ experiences indicated that the patients had been having a hard time because of being abandoned by the family, which would lead to separation anxiety. One participant said: “ … being away from the family is hard for them …” *(p*_*14*_*).* The participants’ experiences also showed that the communication of nurses with these patients could reduce their social isolation problems, anxiety, and stress. As mentioned by one of the participants: “ … they need a strong connection … , in fact, social isolation greatly torments them ... “ *(p*_*15*_*).*

### Need for quality improvement of services

Data analysis showed that the patients needed to receive high quality care services from health staff. Also, data analysis highlighted the patients’ needs for physical support during the disease course. Patients with underlying disorders needed more attention and special equipment. Under this category, there were four sub-categories of physical care, necessity for nutritional therapy, orientation, and isolating of critically ill patients.

#### Physical care

Data analysis showed that the patients, in terms of the disease severity, needed special attention and support from the health care team. To meet these needs, there are requirements for equipment such as intubation devices, thermometers, medications, etc., as well as health care procedures such as suctioning secretions, catheterization, and other physical care. The participants noted that patients with underlying diseases, who should be taken care of more rigorously, required special attention from the treatment staff. One participant mentioned: “... for example, patients with tracheae have problems in coughing ... they should be suctioned regularly, and on the other hand, their lungs have inadequate function due to the coronavirus ...” *(p*_*17*_*)*, and another participant mentioned: “.... they need to be suctioned .... those with catheters need additional care to prevent urinary tract infections .... it is also needed to change their position every two hours ... “ *(p*_*20*_*).*

#### Necessity for nutritional therapy

Data analysis showed that one of the important needs of COVID-19 patients was paying attention to their nutritional needs. The participants’ experiences showed that these patients develop anorexia due to anxiety, stress, dyspnea, and coughing. Considering the nature of the disease, they need to have a proficient immune system and therefore a rich diet and counseling with nutritionists. One participant stated: “... their nutrition is really important and should be rich ...”*(p*_*20*_*)*, and another participant, referring to patients’ dehydration, said: “.... I see that dehydration agonizes these patients ....” *(p*_*15*_*).*

#### Orientation

The analysis of the participants’ experiences showed that COVID-19 patients should become familiar with the hospital environment and fully informed of their condition within early hours of arrival to the ward. Since nurses’ protective clothes are unfamiliar to the patients, they must become recognizable by writing nurses’ names or posting their photos. The data showed that familiarizing patients with the hospital environment and simply explaining the function of medical equipment can encourage them to follow therapeutic instructions. The participants noted that fear was a major obstacle to treatment, but patients who were familiarized with the environment and equipment had a good compliance with the instructions. In this regard, one of the participants mentioned: “.... protective clothes surely have an effect on the quality of health care.... most patients don’t even know if I’m a man or a woman ...” *(p*_*15*_*).* Another participant stressed on the importance of familiarizing patients with equipment and its effect on their compliance with the instructions: “.... when I explained to the patient that how the monitor worked, he had a constant eye on it, and whenever his saturation would rise above 90, he would feel calm ... “ *(p*_*16*_*).*

#### Necessity for isolating of critically ill patients

Data analysis showed that being witnessed to the death or aggravating condition of others would cause fear and anxiety and disrupt other patients’ hemodynamic status. The experience of the participants indicated that critically ill patients should be separated from the patients with a moderate-severe disease. One of the participants explained his experience regarding the adverse effects of one patient’s death on others’ spirit: “.... when one of the patients expired, others were frightened thinking that they would be the next... seeing the death of another patient, some patients experienced the fluctuation of blood pressure or a reduction in blood sugar .... “ *(p*_*17*_*).*

### Need for upgrading of information

Data analysis showed that most COVID-19 patients were unaware of the disease’s dimensions and did not follow the principles of disease prevention. This category was divided into two subcategories: “the necessity for improving awareness and fighting superstition” and “institutionalizing a disease-prevention culture”.

#### Necessity for improving awareness and fighting superstition

Analyzing the data suggested that people have inadequate knowledge about the COVID-19 disease, and in some cases, they have misleading and somehow superstitious beliefs. The participants noted that the patients may delay referring to hospitals due to the lack of awareness and a fear of the disease. One of the participants quoted a patient: “... is it true if one is infected by the virus, they inject a drug to kill him? “ … these superstition beliefs are present among some people ... “*(p*_*14*_*).*

#### Institutionalizing a disease prevention culture

Data analysis indicated that some patients still did not believe in preventive actions and following the disease prevention protocols. In this regard, one of the participants, with respect to patients’ cultural beliefs and the necessity of observing social distancing, said: “ … I have a feeling that our social culture is relatively weak in this area. We go to a patient’s bedside and tell him to put on his mask … this would make him upset .... there should be a culture of self-awareness and self-care ....” *(p*_*15*_*).*

### Need for improving of social support

Humans are social beings and interested to be in a community and communicate with others. Data analysis showed that in order to provide care for COVID-19 patients, special attention is required with respect to social support so that the patients feel less homesick and lonely during this period. In this category, there were two sub-categories of the provision of familial communications and personal accessories.

#### Provision of familial communications

Based on the data analysis, one of the patients’ problems was the lack of familial support. The patients needed to communicate with their families and relatives during isolation and hospitalization. The participants verified that phone or video communications of the patients with their family members created a psychological peace for them and positively affected their recovery process. One of the participants said, “... they need so much psychological support... for example, an old mother felt very well as soon as she saw her son from a distance....” *(p*_*17*_*).* Referring to the patients’ reluctance to treatments, one participant said: “... most of our patients whose families did not come were almost disappointed … for example, they would not take their pills and resist treatment .... “*(p*_*16*_*).*

#### Provision of personal accessories

An analysis on the participants’ experiences revealed that COVID-19 patients would like to use their personal belongings during hospitalization. The nurses noted that providing them with their personal belongings could lead to psychological and mental calm. One of the participants said, “... we had a patient that said she would like to drink tea with her own flask .... while she was very anxious and fastidious and had sleep problems at the nights before … when I brought him the flask, she barely drank a half cup of tea, felt calm, and slept all the night... “*(p*_*16*_*).*

### Need for spiritual care

The data analysis showed that one of the patients’ needs was to pay attention to their spiritual needs. The participants’ experiences showed that listening to prayers gave the patients mental peace and a pleasant feeling. Also, the participants recalled that the patients were influenced by verses from the Holy Quran and prayed for themselves and other patients. One of the participants said about the necessity of paying attention to the patients’ spiritual dimension: “.... patients were asking us to pray for them. Sometimes, we were praying together ...” *(p*_*17*_*).*

### Need for social welfare

Data analysis showed that economic problems were among the main concerns of COVID-19 patients. The participants mentioned that some of the patients were constantly thinking of economic issues, and this would create a great deal of stress, affecting the course of their illness. One of the participants said: “ … we had an economically poor patient who was constantly worried about his household and financial issues... what is my family doing now? The income that I was responsible for has actually been cut … all these issues bothered him a lot ... “*(p*_*15*_*).*

## Discussion

The current study was conducted to explore nurses’ perception of caring needs of the patients with COVID-19. The data analysis showed that COVID-19 patients were psychologically, physically, socially, economically, and spiritually affected by the disease. Therefore, they should be comprehensively supported by medical staff and other supporting systems.

In the present study, the fear of death was reported to be a stressful and annoying factor for the patients. Fear can lead to behavioral disorders and severe psychological reactions including suicide [14]. It has been estimated that the suicide rate due to the fear of COVID-19 will surge the next year, and because of this, an interventional plan has been implemented in the United States [15]. The fear of death not only predicts COVID-19 anxiety but also plays a causal role in different mental health conditions. So, it has been noted that mental health programs should focus on directly addressing death anxiety in these patients. It seems that cognitive behavioral therapy can reduce death anxiety [16], and it is recommended that this approach be considered for COVID-19 patients. Further research is essential to determine whether or not treatment for death anxiety improves long-term outcomes and prevents further disorders in vulnerable populations [16].

In this study, findings showed that COVID-19 patients were concerned about society’s view on them and feared of being socially rejected. In fact, being rejected by society and the social stigma have been reported among major concerns of patients during epidemics, particularly in the case of COVID-19 pandemic [17]. During the outbreak of the novel coronavirus, lockdown and communicational limitations were implemented by the aid of military forces in most countries across the world. This phenomenon can strengthen the impression of social stigma and exacerbate social inequalities [18]. The ability of social compatibility actually presents itself during an epidemic [19]. Improving awareness, preventing fake information, and implementing social equality policies (such as equal access to diagnostic and therapeutic facilities) are among the measures that can be helpful to reduce social stigma [20, 21]. Understanding the fact that people who die of COVID-19 are buried without formal funerals aggravates the anxiety and fear of the disease. These findings clearly highlight the needs of COVID-19 patients for psychological interventions which should be taken seriously and incorporated in hospitals’ therapeutic protocols. In this regard, the findings of other studies indicate that focusing on the etiology and epidemiological characteristics of the disease in social networks generally aggravates the public concerns and changes their knowledge and attitude toward COVID-19 [22]. The level of misinformation on Twitter has been alarming, and there is a need for interventional measures to resolve this issue [17].

The present study showed that frustration was a major problem in COVID-19 patients, and the fact that they needed emotional support as a primary requirement. This finding was consistent with the evidence showing a surge in fear and anxiety during diseases’ outbreaks [23]. Lee et al. (2020) found that people with anxiety and stress were more likely to experience frustration, mental crisis, suicidal ideation, and alcohol and substance abuse [24]. Due to the importance of emotional needs and the outcomes of psychological despair, it is recommended to not only focus on clinical symptoms, but also consider psychological counseling and screening programs to identify the patients who are at the early stages of anxiety and despair.

In this study, the findings showed that COVID-19 patients needed to receive high-quality health services. It was found that the patients also had special care needs such as suctioning pulmonary secretions, frequent checking on vital signs, and ventilation. Patients with COVID-19 represent symptoms such as cough, dyspnea, fever, sore throat, and sometimes other nonspecific presentations; nevertheless, only 5% of these patients will require ventilation [25, 26]. In patients with severe symptoms, in addition to ventilation, monitoring and maintaining the function of several vital organs such as the heart and kidneys, as well as extremities (legs and fingers) are very important. Actually, patient-specific medical decisions and interventions are necessary in these scenarios [27]. In line with the findings of this study, other researchers have also emphasized on promoting and monitoring the quality of intensive care [25] and the regular updating of health care guidelines [28] for these patients.

Findings in this study showed that the patients with COVID-19 may develop anorexia nervosa due to the fear and anxiety of the disease. Therefore, there is a critical need for paying attention to the nutritional status of these patients. The importance of this observation has been reiterated in several studies [29–31]. Improper nutrition can weaken the immune system, promote chronic inflammation, and finally disrupt the host’s defense against viruses. The COVID-19-induced inflammation and neurological dysfunction may deteriorate and progress to long-term consequences such as dementia and neurological diseases in those with unhealthy diets [30]. In addition to providing a healthy diet for these patients, educating the public should also be one of the main priorities of health systems to encourage individuals to employ healthy eating habits and choose appropriate regimens to prevent COVID-19 long-term effects.

In line with the findings of the current study, another report showed that anxiety and a feeling of loneliness, as major consequences of COVID-19, relieved after communicating with friends and family members via social media [32]. The findings of other studies also showed that social support is an important factor in reducing stress during outbreaks [33, 34]. The COVID-19 has multiple unknown dimensions, and in the present study, we noticed an immediate need for boosting the patients’ awareness of the disease. In addition to inadequate knowledge, improper perceptions about the disease are among the issues that we should be focusing on. In line, misinformation about some aspects of the disease has been noted as a point of concern in other studies [35]. In fact, misinformation on the coronavirus outbreak has become a global crisis in a way that the popularity of unconfirmed information sources has surpassed that of the World Health Organization (WHO) and the Centers for Disease Control (CDC) [36]. This phenomenon can predispose to psychosocial disorders, and therefore it is essential to increase the public health literacy, monitor social media, and activate public health organizations in social networks to create transparency and boost confidence in governments and non-governmental agencies.

Our findings showed that one of COVID-19 patients’ needs was taking into account their spiritual dimensions. Spiritual care can effectively reduce stress and augment the feelings of wellness and integrity, as well as interpersonal relationships among patients [37]. It seems that spiritual care is one of the missing items of caring protocols for these patients. As these programs can be very helpful, it is recommended to use a team of psychologists and religious experts in order to provide a comprehensive care for these patients [38].

The mental entanglement of hospitalized COVID-19 patients with economic issues was another finding of the present study. Reduced financial trades and monetary activities [39], declined production of manufacturers, and recessions in tourism, food, education, and oil industries are inevitable during pandemics. A reduction in workforce is another major consequence amid the novel coronavirus outbreak, raising concerns about a global economic crisis [40]. So, immediate measures are needed to alleviate the economic burden of the disease (e.g. lost jobs) in vulnerable social groups.

One of the limitations of this study was that we only focused on nurses’ perspectives; however, the views of patients with COVID-19 could also provide richer information. Therefore, it is suggested to explore COVID-19 patients’ perspectives on the disease. Regarding the urgency of the situation and the fact that the data needed to be collected in the shortest time possible, the performance of validation procedures was compromised. However, the validity of the data was ensured using alternative methods. Another limitation of this study was conducting the interviews via phone calls to reduce the risk of disease transmission for both the interviewee and interviewer. This could have prevented a deep understanding of the phenomenon; nevertheless, the researchers tried to make the phone calls as deep and effective as possible.

## Conclusion

The aim of this study was to explore nurses’ perception about the care needs of COVID-19 patients. The data showed that these patients were psychologically, physically, socially, economically, and spiritually affected by the disease, highlighting the need for a comprehensive care by medical staff and other supporting systems. Factors such as death anxiety, taboo disease, frustration, and social isolation cause stress and anxiety in the patients, which can be resolved by continuous psychological counseling and providing spiritual care during the disease’s course from the onset of symptoms to a few days after recovery. The COVID-19 patients experience various physical symptoms such as pain, fever, dyspnea, and cardiovascular and nutritional problems, etc. which all need to be addressed by medical teams. The lack of knowledge about the various dimensions of the disease, superstition beliefs, and low compliance with preventive measures indicate an urgent need for improving the public knowledge about the disease. Considering the economic problems and the ensuing global recession, governments along with non-governmental organizations (NGOs) and charities should identify poor and vulnerable patients and endeavor to reduce their problems.

## Data Availability

The datasets generated during and/or analyzed during the current study are available from the corresponding author on request.

## Abbreviations

NGOs: Non-governmental Organizations
CDC: Centers for disease control and prevention
WHO: World health Organization
